# Importance of Trade

**Published:** 1872-01

**Authors:** 


### IMPORTANCE OF TRADE.

Of the fifteen thousand occupants of the penitentiaries of thirty States of the Union, seventy-seven men out of each hundred, more than three fourths of the whole number, had not learned a trade, showing how much of truth was in the assertion of Franklin, harsh as it may seem to some: The man who allows his son to grow up without a trade or a profession by which he can honorably support himself in case of misfortune and reverses, teaches that son how to become a thief. More than that has become true since Franklin's time. Our country has become greatly more crowded, in proportion, the strife for bread becomes more and more a struggle for life, and in view of such a fact, the parent who does not provide for his daughter a certain income every year as long as she lives, or fails to educate her to some calling by which she could earn a living by her own exertions, runs a fearful risk of leaving that daughter to a life of want and destitution and infamy.

